# High Prevalence of Macrolide-Resistant *Bordetella pertussis* and *ptxP1* Genotype, Mainland China, 2014–2016

**DOI:** 10.3201/eid2512.181836

**Published:** 2019-12

**Authors:** Lijun Li, Jikui Deng, Xiang Ma, Kai Zhou, Qinghong Meng, Lin Yuan, Wei Shi, Qing Wang, Yue Li, Kaihu Yao

**Affiliations:** Beijing Pediatric Research Institute, Beijing Children’s Hospital, Capital Medical University, Beijing, China (L. Li, Q. Meng, L. Yuan, W. Shi, Q. Wang, Y. Li, K. Yao);; Shenzhen Children’s Hospital, Shenzhen, China (J. Deng);; Jinan Children’s Hospital, Shandong University, Shandong, China (X. Ma);; Nanjing Children’s Hospital, Nanjing Medical University, Nanjing, China

**Keywords:** Bordetella pertussis, antibiotic sensitivity, antimicrobial sensitivity, antimicrobial resistance, macrolide resistance, virulence-related genotype, *ptxP1*, bacteria, China

## Abstract

According to the government of China, reported cases of pertussis have increased remarkably and are still increasing. To determine the genetic relatedness of *Bordetella pertussis* strains, we compared multilocus variable-number tandem-repeat analysis (MLVA) results for isolates from China with those from Western countries. Among 335 isolates from China, the most common virulence-associated genotype was *ptxA1/ptxC1/ptxP1/prn1/fim2–1/fim3A/tcfA2*, which was more frequent among isolates from northern than southern China. Isolates of this genotype were highly resistant to erythromycin. We identified 36 *ptxP3* strains mainly harboring *ptxA1* and *prn2* (35/36); *ptxP3* strains were sensitive to erythromycin and were less frequently from northern China. For all isolates, the sulfamethoxazole/trimethoprim MIC was low, indicating that this drug should be recommended for patients infected with erythromycin-resistant *B. pertussis.* MLVA of 150 clinical isolates identified 13 MLVA types, including 3 predominant types. Our results show that isolates circulating in China differ from those in Western countries.

Whooping cough (pertussis) is a highly contagious respiratory disease mainly transmitted by aerosolized respiratory droplets. The causative agent is a gram‐negative bacterium first reported in 1906 and later named *Bordetella pertussis*.

The gradual introduction of whole-cell pertussis vaccines (WCV) worldwide in the mid-1940s (*1*) was followed by a dramatic decrease in illness and death from pertussis. In China, pertussis immunization with WCV was introduced in the early 1960s (*2*). The vaccine is administered as part of a trivalent combined vaccine during the first year of life, at months 3, 4, and 5. Since 1982, many countries have recommended also giving a booster dose to children at 18–24 months of age (*3*,*4*). However, the side effects and safety of WCV aroused considerable public concern globally, which stimulated the introduction of acellular pertussis vaccines (ACV). In most developed countries, the shift from WCV to ACV was implemented in the 1990s and the early 2000s. In China, both WCV and ACV have been used since 2006 (*5*) and ACV alone since 2013 (*2*). The ACV used in China was made by co-purifying techniques and mainly contained pertussis toxin and filamentous hemagglutinin, as well as a few other antigens that cannot be completely removed.

In the 1990s, however, pertussis began to reemerge in several highly immunized populations, and the number of pertussis cases is still increasing worldwide (*6*). According to the Chinese Center for Disease Control and Prevention (*7*), reported pertussis cases increased substantially after widespread vaccination with ACV and are still increasing (Figure 1). Many factors have contributed to the increase: improved diagnostics, increased awareness, waning immunity, and pathogen adaptation. We aimed to determine molecular evolution and pathogen adaptation of *B. pertussis.*

**Figure 1:**
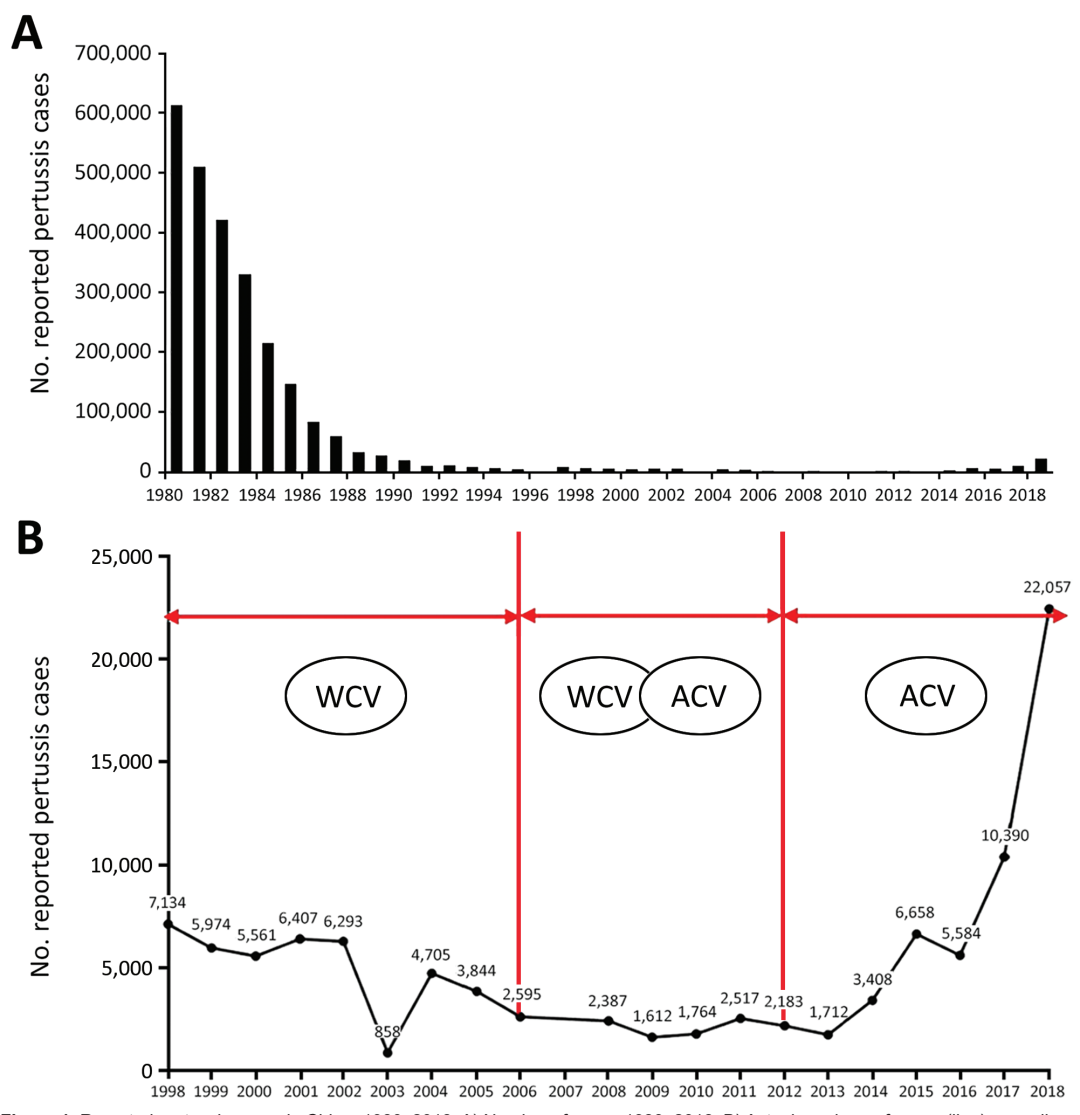
Reported pertussis cases in China, 1980–2018. A) Number of cases 1980–2018. B) Actual numbers of cases (line) according to vaccine type administered during a given period, 1998–2018. ACV, acellular pertussis vaccine; WCV, whole-cell pertussis vaccine.

Macrolides have been used to treat and prevent whooping cough for ≈50 years, but there have been multiple reports of erythromycin resistance (*3*,*8*,*9*). Our previous study in northern China showed a strikingly high rate of macrolide resistance (91.9%) in *B. pertussis* (*2*). Whether the high erythromycin resistance rate was also widespread across mainland China or whether it was a temporary epidemic remains unknown. In this study, we recovered 335 *B. pertussis* isolates from patients in mainland China and investigated their susceptibility to erythromycin and other antimicrobial drugs. Our goal was to provide effective treatment guidance in the face of erythromycin resistance, because although sulfonamides are the second-line treatment, their use in infants <2 months of age is prohibited. To discern the population structures of *B. pertussis* isolates in China, we also investigated the distribution of virulence-related genotypes by using antigen genotyping for all 335 isolates. To determine the genetic relationship between strains, we then performed multilocus variable-number tandem-repeat analysis (MLVA) on a subset of 150 isolates. 

## Methods

### Bacterial Strains, Patient Demographics, and Clinical Information 

From October 2014 through December 2016, all patients suspected to have pertussis were routinely examined by culture of nasopharyngeal swab samples. We included in our study those patients for whom cultures were positive for *B. pertussis*. We analyzed 335 *B. pertussis* isolates collected from patients in 4 cities in China: 245 isolates from Beijing Children’s Hospital, 21 from Jinan Children’s Hospital, 13 from Nanjing Children’s Hospital, and 56 from Shenzhen Children’s Hospital. We recorded demographic data (e.g., sex, age, and residential address) for all patients with confirmed pertussis. We also recorded clinical information, available for 109 patients at Beijing Children’s Hospital (e.g., vaccine history, coughing of family members during the patient’s incubation period, leukocyte count, lymphocyte ratio, budesonide aerosol inhalation, and antimicrobial drug use before swab collection). Parents or legal guardians provided informed written consent before nasopharyngeal swab samples were collected. This study was reviewed and approved by the ethics committee of each hospital.

### Bacterial Culture

We plated all nasopharyngeal specimens onto charcoal agar (Oxoid; ThermoFisher Scientific, https://www.thermofisher.com) supplemented with 10% defibrinated sheep blood and *Bordetella* selective supplement SR0082E (cephalexin) and incubated the plates at 35–37°C for 3 days. We confirmed suspected *B. pertussis* colonies by the slide agglutination test with *B. pertussis* and *B. parapertussis* antiserum (Remel; ThermoFisher Scientific, http://www.thermofisher.com). We stored all isolates at −80°C until further analysis.

### Antimicrobial Susceptibility Testing

We determined antimicrobial susceptibility by E-test and Kirby-Bauer disk diffusion. Before susceptibility testing, we thawed bacterial preservation tubes from a −80°C freezer and performed culture at 35–37°C on the charcoal agar plates containing 10% sheep blood for 72 h. We then subcultured bacterial suspension with 0.5 McFarland turbidity standard on 25 mL charcoal agar containing 10% sheep blood in a 90-mm diameter culture dish. We used the E-test to determine susceptibility to erythromycin, clindamycin, amoxicillin, ampicillin, ceftriaxone, levofloxacin, sulfamethoxazole/trimethoprim, amikacin, clarithromycin, azithromycin, doxycycline, and aztreonam. Because of a lack of adequate E-test strips for some antimicrobials, we tested some isolates against several antimicrobials according to sequential order without any initial selection criteria (i.e., 310 isolates for doxycycline, 222 isolates for amikacin, 86 isolates for aztreonam, 83 isolates for clarithromycin, and 83 isolates for azithromycin). We measured MICs and inhibition zone sizes when the plates were incubated for 4 days. The Clinical and Laboratory Standards Institute and the European Committee on Antimicrobial Susceptibility Testing do not yet provide breakpoint criteria for antimicrobial susceptibility for *B. pertussis*. We report 50% MICs, 90% MICs, and MIC ranges. We also tested susceptibility to erythromycin with Kirby-Bauer disk diffusion, and according to some studies, an inhibition diameter >42 mm suggested complete susceptibility to erythromycin. MICs <0.12 mg/L are considered susceptible to erythromycin (*8*). For quality control strains, we included *Haemophilus influenzae* ATCC49247 and *Staphylococcus aureus* ATCC30913 in each batch of susceptibility tests.

### Genotyping

We extracted the genomic DNA of isolates by using a DNA extraction kit (SBS Genetic Co. Ltd, http://www.sbsbio.com) according to the manufacturer’s instructions. We amplified and sequenced the 7 genes of *B. pertussis* isolates (*ptxA, ptxC, ptxP, prn, fim2, fim3, and tcfA2*) as previously described (*2*) and identified genotypes by comparison with designated alleles in GenBank.

### Sequencing of the *B. pertussis* 23S rRNA Gene 

The A2047G mutation has been proven to be the cause of erythromycin resistance of *B. pertussis* (*8*). We amplified and sequenced domain V of the 23S rRNA gene of 335 isolates as previously described (*2*). We then compared the sequences with the X68323 sequence and the allele of *B. pertussis* Tohama I strain (accession no. NC_002929.2) in GenBank.

### MLVA

We performed MLVA according to the procedures described by Schouls et al (*10*). We used 5 variable-number tandem-repeats (VNTRs) to characterize 150 randomly selected clinical isolates, which were from 6 geographic areas in China (i.e., 83 from northern, 33 southern, 26 eastern, 3 central, 4 northeastern, and 1 northwestern). We included reference strains (Tohama I) with known MLVA types as positive controls in each run and expressed the results as MLVA types. We assigned MLVA types by using the *B. pertussis* MLVA database (https://www.mlva.net); the assignment of MLVA types was based on the combination of repeat counts for VNTR1, VNTR3a, VNTR3b, VNTR4, VNTR5, and VNTR6. We generated minimum spanning trees from the 6 MLVA loci by using BioNumerics version 7.6 (Applied Maths, http://www.applied-maths.com).

### Statistical Analyses

We used SPSS 17.0 (https://www.ibm.com/analytics/spss-statistics-software) for statistical analyses and analyzed the data by using χ^2^ or Fisher exact tests, as appropriate. We considered p<0.05 to be significant.

## Results

### Patient Demographic and Clinical Data

**Figure 2:**
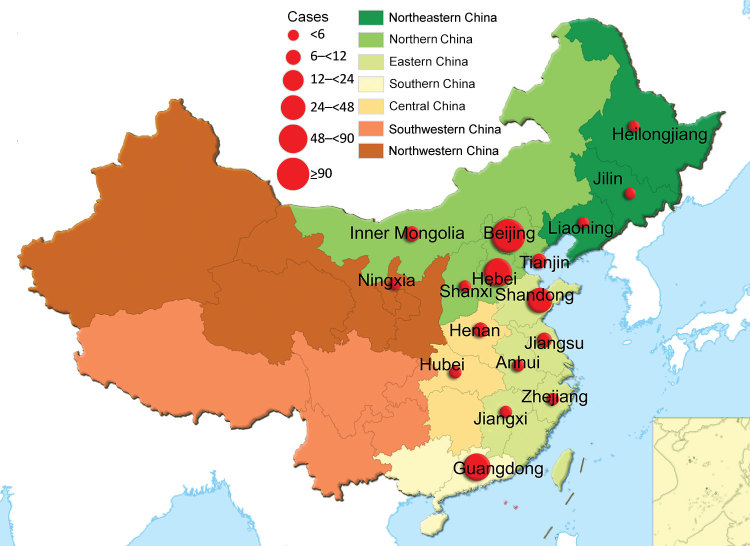
Geographic distribution of 335 patients with pertussis diagnosis, China, 2014–2016. Colors indicate different administrative regions. Red circles indicate numbers of patients; the larger the circle, the more patients in the province.

The 335 patients (189 male and 146 female) were from Beijing (n = 101), Hebei (n = 91), Guangdong (n = 56), Shandong (n = 30), Jiangsu (n = 10), Tianjin (n = 9), Inner Mongolia Autonomous Region (n = 7), Henan (n = 6), Shanxi (n = 5), Anhui (n = 5), Jiangxi (n = 4), Zhejiang (n = 3), Heilongjiang (n = 2), Jilin (n = 2), Liaoning (n = 2), Hubei (n = 1), and Ningxia (n = 1). These provinces and municipalities covered 6 geographic areas of China defined by the government. In this study, 213 patients were from northern, 56 southern, 52 eastern, 7 central, 6 northeastern, and 1 northwestern China (Figure 2).

The age distribution of the 335 patients was as follows: 119 (35.5%) were <3 months of age; 113 (33.7%), 3–5 months; 80 (23.9%), 6–18 months; 11 (3.3%), 19 months–2 years; and 12 (3.6%), 3–12 years. Of the 335 patients, 195 were not vaccinated, 32 had received 1 dose of pertussis vaccine, 16 had received 2 doses, and 26 had received 3 or 4 doses. The vaccination history of 66 patients could not be confirmed. For 109 patients, we reviewed hospital medical records carefully for more comprehensive clinical information (Table 1).

**Table 1:** Clinical characteristics of 109 pertussis patients, Bejing Children’s Hospital, Beijing, China, October 2014–December 2016*

Patient characteristic	No. (%) patients			p value
	Total patients, n = 109	Fully vaccinated, n = 17	Not vaccinated or undervaccinated, n = 92	
Fever	28 (25.69)	6 (35.29)	22 (23.9)	0.32
Rhinorrhea	47 (43.12)	12 (70.59)	35 (38.0)	0.01
Nasal congestion	50 (45.87)	8 (47.06)	42 (45.65)	0.92
Purulent nasal secretion	5 (4.59)	1 (5.88)	4 (4.35)	1.00
Cough				
Paroxysmal	90 (82.57)	13 (76.47)	77 (83.70)	0.71
Spasmodic	95 (87.16)	13 (76.47)	82 (89.13)	0.30
Whooping	46 (42.20)	9 (52.94)	37 (40.22)	0.33
Excessive phlegm	48 (44.04)	5 (29.41)	43 (46.74)	0.19
Vomiting	66 (60.55)	9 (52.94)	57 (61.96)	0.49
Cyanosis	72 (66.06)	11 (64.71)	64 (69.57)	0.83
Apnea	27 (24.77)	4 (23.53)	20 (21.74)	1.00
Convulsion	1 (0.92)	0	1 (1.09)	1.00
Sweats	25 (22.94)	7 (41.18)	18 (19.57)	0.05
Subconjunctival hemorrhage	4 (3.67)	0 (0.00)	4 (4.35)	0.50
Ulcer of lingual frenum	1 (0.92)	0	1 (1.09)	0.85
Leukocytosis				
>10 × 10^9^ cells/L	74 (67.89)	6 (35.29)	68 (73.91)	<0.01
>60 ×10^9^ cells/L	2 (1.89)	0	0	NA
Lymphocytosis†	73 (66.97)	8 (47.06)	65 (70.65)	0.06
Antimicrobial drugs prescribed before culture				
Erythromycin	66 (60.55)	7 (41.18)	59 (31.52)	NA
Cephalosporin	77 (70.64)	11 (64.71)	66 (71.74)	NA
Azithromycin	16 (14.68)	4 (23.53)	13 (14.13)	NA
Amoxicillin	8 (7.34)	2 (11.76)	6 (6.52)	NA
Clarithromycin	2 (1.83)	0	0	NA
Mezlocillin	1 (0.92)	0	0	NA
Imipenem	1 (0.92)	0	0	NA
Aztreonam	1 (0.92)	0	0	NA
Household contacts	52 (47.71)	0	0	NA
Culture-based diagnosis for household contacts‡	4 (3.67)	0	0	NA
Reexamination of culture§	4 (3.67)	0	0	NA

*NA, not analyzed. †Lymphocyte:total leukocyte ratio 66.47% ± 11.47%.  ‡Bacterial culture results of the 4 household contacts were negative. §Reexamination of bacterial culture of 4 patients performed 2 weeks later produced negative results.

### Antimicrobial Susceptibility and 23S rRNA Gene Mutations

All 292 isolates with an erythromycin MIC >256 mg/L showed a 6-mm inhibition zone diameter on Kirby-Bauer disk diffusion (Table 2); all of these isolates had the A2047G mutation in the 23S rRNA gene. The remaining 43 isolates had an erythromycin MIC <0.125 mg/L, of which only 2 had a MIC of 0.125 mg/L. The diameter of the erythromycin disk was >42 mm for 42 isolates and 36 mm for 1 isolate. Isolates with an erythromycin MIC >256 mg/L had MICs >256 mg/L each for clindamycin, clarithromycin, and azithromycin. The MIC range for sulfamethoxazole/trimethoprim was low (0.002–0.5 mg/L) (Table 1). The proportions of isolates resistant to erythromycin in northern China (194/213) and southern China (35/56) differed significantly (χ^2^ = 28.6; p<0.001).

**Table 2:** Antimicrobial susceptibility test results for *Bordetella pertussis* isolates in study of prevalence of macrolide-resistant *B. pertussis* and *ptxP1* genotype, mainland China, 2014–2016*

Drug	No. isolates	E‐test, mg/L				Kirby-Bauer disk diffusion	
		MIC_50_	MIC_90_	MIC range		Range of inhibition zone, mm	Rate of susceptibility, %†
Erythromycin	335	>256	>256	0.032 to >256		668	12.5%
Clindamycin	335	>256	>256	0.25 to >256		NT	NT
Amoxicillin	335	0.5	1	0.125 to 2		NT	NT
Ampicillin	335	0.25	0.5	0.032 to 1		NT	NT
Levofloxacin	335	0.5	1	0.064 to 1		NT	NT
Sulfamethoxazole	335	0.064	0.25	0.002 to 0.5		NT	NT
Ceftriaxone	335	0.25	0.5	0.064 to 2		NT	NT
Amikacin	222	8	8	2 to 32		NT	NT
Clarithromycin	83	>256	>256	0.032 to >256		NT	NT
Azithromycin	83	>256	>256	0.016 to >256		NT	NT
Doxycycline	310	8	8	1 to 16		NT	NT
Aztreonam	86	8	32	4 to 32		NT	NT

*NT, not tested in this study. †An inhibition diameter >42 mm suggested that the isolate was susceptible to erythromycin.

### Genotypes

The most common virulence-associated genotype of all *B. pertussis* strains was *ptxA1/ptxC1/ptxP1/prn1/fim2–1/ fim3A/tcfA2;* frequency was 87.2% (292/335) (Table 3). We identified 36 *ptxP3* strains, which mainly harbored *ptxA1* and *prn2* (35/36). The *ptxP3* strains were more frequent in southern than in northern China (16/56 vs. 17/213; χ^2^ = 17.5; p*<*0.001).

**Table 3:** Genotype profiles for 335 *Bordetella pertussis* isolates from mainland China, 2014–2016

Genotype profile*	No. (%) isolates							
	Total					Region		
		Year				Northern China	Southern China	Other†
		2014	2015	2016				
*ptxA1/ptxC1/ptxP1/prn1/fim2–1/fim3A/tcfA2*	5 (1.5)	0	3 (1.7)	2 (1.5)		2 (0.9)	3 (5.4)	0
*ptxA1/ptxC2/ptxP1/prn1/fim2–1/fim3A/tcfA2*	1 (0.3)	0	1 (0.6)	0		1 (0.5)	0	0
*ptxA1/ptxC1/ptxP1/prn3/fim2–1fim3A/tcfA2*	1 (0.3)	0	1 (0.6)	0		0	1 (1.8)	0
*ptxA1/ptxC2/ptxP3/prn9/fim2–1/fim3A/tcfA2*	1 (0.3)	0	0	1 (0.7)		0	1 (1.8)	0
*ptxA1/ptxC2/ptxP3/prn2/fim2–1/fim3B/tcfA2*	2 (0.6)	1 (3.7)	1 (0.6)	0		2 (0.9)	0	0
*ptxA1/ptxC1/ptxP3/prn2/fim2–1/fim3A/tcfA2*	5 (1.5)	1 (3.7)	3 (1.7)	1 (0.7)		5 (2.3)	0	0
*ptxA1/ptxC2/ptxP3/prn2/fim2–1/fim3A/tcfA2*	28 (8.4)	0	8 (4.7)	20 (14.7)		10 (4.7)	15 (26.8)	3 (4.5)
*ptxA1/ptxC1/ptxP1/prn1/fim2–1/fim3A/tcfA2*	292 (87.2)	25 (92.6)	155 (90.1)	112 (82.4)		193 (90.6)	36 (64.3)	63 (95.5)
Total	335 (100)	27 (100)	172 (100)	136 (100)		213 (100)	56 (100)	66 (100)

*Genotype of vaccine strain in China: *ptxA2/ptxC1/ptxP1/prn1/fim2–1/fim3A/tcfA2.* †Northeastern, northwestern, eastern, and central southern China.

All *ptxP3* strains had lower MICs for erythromycin (0.023–0.125 mg/L) and clindamycin (0.094–4 mg/L). The isolates with erythromycin MIC >256 mg/L were all typed as *ptxP1*.

### MLVA Combined with Virulence-Associated Genotyping and the A2047G Mutation of 23S rRNA

**Figure 3:**
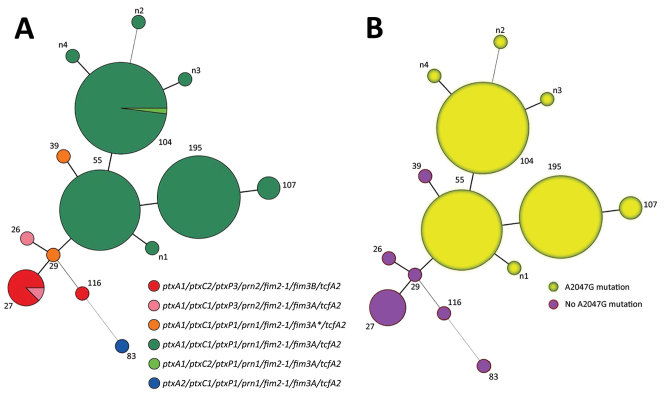
Minimum spanning tree of multilocus variable-number tandem-repeat analysis (MLVA) types of 150 *Bordetella pertussis* isolates collected in China, 2014–2016. Each circle represents an MLVA type, with the number next to the circle. Circle sizes are proportional to the number of isolates belonging to the particular MLVA type. A) Allelic profiles. Circle colors indicate the diﬀerent allelic profiles of vaccine antigen genes and different erythromycin sensitivities. B) Presence or absence of A2047G mutation.

The 150 isolates typed by MLVA were divided into 13 MLVA types: MT26, MT27, MT29, MT39, MT55, MT104, MT107, MT116, MT195, and 4 new types (N1–N4). The major MLVA types were MT104, MT55, and MT195. Both MT55 and MT195 have a uniform allelic profile: *ptxA1/ptxC1/ptxP1/prn1/fim2–1/ fim3A/tcfA2*. MT104 has 2 profiles; 1 profile was the same as that of isolates of MT55 and MT195, and another is *ptxA1/ptxC2/ptxP1/prn1/fim2–1/fim3A/tcfA2*, which differs only in *ptxC* (Figure 3). The genotypes of MT29 isolates (*ptxA1/ptxC1/ptxP1/prn1/fim2–1/fim3A/tcfA2*) differed from MT 55 isolates only in *fim3*. Eight isolates of MT27 had 2 profiles that differed in *ptxC* and *fim3*, *ptxA1/ptxC1/ptxP3/prn2/fim2–1/fim3A/tcfA2*, and *ptxA1/ptxC2/ptxP3/prn2/fim2–1/ fim3B/tcfA2*. The genotypes of MT26 (*ptxA1/ptxC1/ptxP3/prn2/fim2–1/ fim3A/tcfA2*) were the same as one of the profiles of isolates of MT27. Although isolates with the same genotype profiles varied in MLVA type, these types were similar for isolates with similar virulence-related genotypes (Figure 3, panel A). Overall, all isolates of MT26, MT27, and MT116 carried *ptxP3/ prn2*, and all isolates of MT55, MT104, and MT195 carried *ptxP1/ prn1*. All isolates of MT55, MT104, MT195, and MT116 had the A2047G mutation of 23S rRNA; no isolates of MT26, MT27, MT29, or MT116 had this mutation (Figure 3, panel B). The MLVA types of isolates with the mutation at the 2047 site of 23S rRNA were closer to each other, and those without the mutation were also linked to each other except for MT39 (Figure 3, panel B).

## Discussion

**Figure 4:**
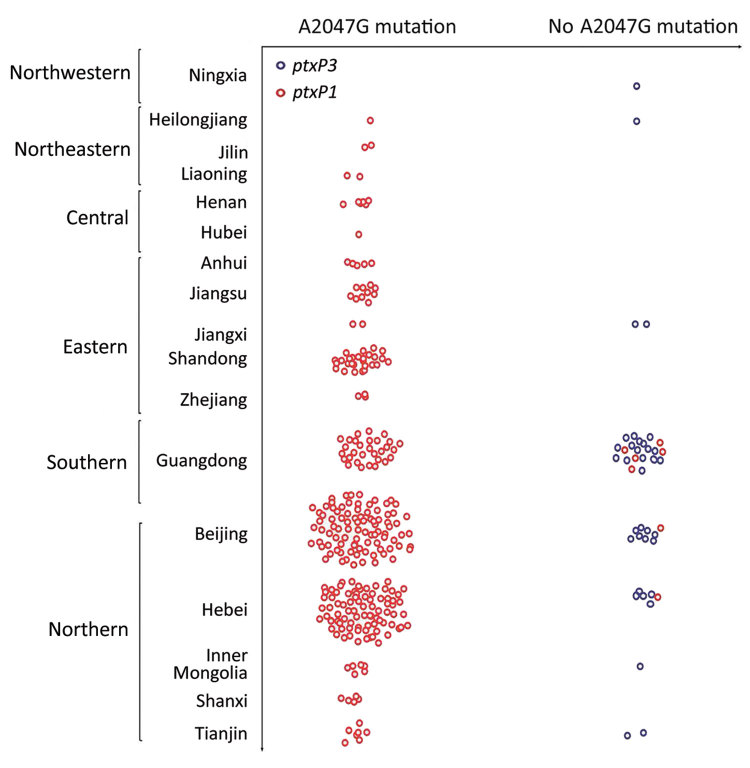
Geographic differences in frequency of erythromycin resistance in *Bordetella pertussis* isolates, China, 2014–2016.

The preferred treatment for persons with pertussis is erythromycin or another macrolide. The first reports of erythromycin*-*resistant *B. pertussis* in the United States were published in 1994 (*11*). Since then, and not only in the United States, several erythromycin-resistant *B. pertussis* isolates have been reported (*12**,**13*), but no evidence of an epidemic of erythromycin-resistant pertussis occurred in any other country except China. In 2014, a study in Xi’an, China, detected high prevalence of erythromycin-resistant *B. pertussis;* 85% (85/100) of strains had the A2047G mutation (*5*). In our previous study, the *B. pertussis* isolates from the 1970s and 2000–2008 were susceptible to macrolides, and 91.9% of isolates collected during 2013–2014 were resistant to macrolides (MIC >256 mg/L) (*2*). However, our previous study was performed only at Beijing Children’s Hospital, and the number of samples was limited. In the current study, we found that erythromycin-resistant *B. pertussis* strains caused infection in each of the 6 areas in China; 87.5% (292/335) of isolates were resistant to erythromycin (MIC >256 mg/L). In 2003, Bartkus et al. confirmed that the 23S rRNA A2047G mutation was a mechanism of erythromycin resistance to *B. pertussis* (*8*). All 292 erythromycin-resistant *B. pertussis* isolates in our study had the 23S rRNA A2047G mutation, a finding that is consistent with other reports. The erythromycin-resistant *B. pertussis* strains were isolated from children in different districts (Figure 4), which means that erythromycin-resistant *B. pertussis* is spread widely across China.

We also found geographic differences in erythromycin resistance among *B. pertussis* isolates. Among those from northern China, the rate reached up to 91.1% (194/213), in accordance with our previous study in 2013–2014 (91.9%, 91/99) (*2*). However, the rate was only 64.3% (36/56) among isolates from southern China. This finding might be associated with differences in antimicrobial drug use between northern and southern China; however, we could not make this comparison because we did not have detailed clinical data for cases in southern China. Because the resistance was closely associated with the *ptxP1* genotype, a geographic difference in *ptxP* genotypes was also found between northern and southern China (Figure 4). Thus, another possible reason for this regional difference is population mobility. Shenzhen is near Hong Kong and Macau, in the most dynamic and developed region in China in terms of economy. Because daily movement of the population in Shenzhen, Hong Kong, and Macau is large and has become the regular lifestyle, the *ptxP3* strains could be imported more frequently. Shanghai, another economic development area, also had a high proportion of *ptxP3* strains (*14*). The *ptxP3* strains could also be transmitted widely because of less macrolide abuse in southern China.

To better determine the genetic diversity of isolates of different genotypes and erythromycin sensitivities, we analyzed 150 clinical isolates by MLVA type. Compared with other findings for China (*9*,*15*), our findings showed that the predominant MLVA types of recent isolates were distinct from strains isolated before widespread vaccination with ACV. Some MLVA types that had been prevalent (MT29 and MT33, frequent in the 1950s; MT294 and MT95, frequent during 1962–1986; and MT91, MT136, and MT152, frequent during 1997–2007) (*15*) had disappeared, and others (MT104, MT55, and MT195) had increased. Unlike trends in other countries (*16*–*18*) that showed that isolates harboring MT27 and MT29 were becoming prevalent over time, isolates from our study were mainly MT55, MT104, and MT195. We found only 8 MT27 isolates and 1 MT29 isolate, which correlates with findings of another study in Xi’an (*9*), in which isolates were collected during 2015–2016 from some parts of northern China. In that study, of 8 MT27 isolates, 5 were from northern China and 3 were from southern China, which was not a significant difference (5/83 vs. 3/22; χ^2^ = 3.26; p = 0.07). MT55, MT104, and MT195 were more frequently isolated in northern China (76/83 vs. 22/33; χ^2^ = 11.17; p*<*0.001).

In our study, the 3 predominant MLVA types (MT104, MT55, and MT195) were closely related, with only 1 difference in the number of VNTR6 allele repeats, suggesting that these types could have evolved from closely related strains. *B. pertussis* isolates harboring MT29 were isolated in the 1950s and 1960s but afterward disappeared, according to another study in China (*15*). Whether MT29 existed from the 1950s or was imported from other countries needs further study. Both MT27 and MT55 were related to MT29, but the difference between MT27 and MT29 was in VNTR3a. VNTR3 and VNTR6 were in the pseudogenes. Although pseudogenes do not encode functional genes, they are key in research of bacterial evolution and dynamic genomes and are associated with expression and regulation of functional genes. 

Our study lays the foundation for further study of whole-genome sequencing to confirm how much and what kind of genetic changes would happen among *B. pertussis* isolates. The great heterogeneity of MLVA types was identified among erythromycin-resistant and erythromycin-nonresistant isolates. All MT104, MT55, and MT195 isolates had the same combination of the virulence-related genotypes, *ptxP1/ptxA1/prn1*, and were resistant to erythromycin; this result corroborates results of another study in which isolates were collected from another northern China city with less population movement (*9*). We found MT27, the most common type in other countries, in only 8 isolates; the virulence-related genotypes were similar to those of isolates from other countries, suggesting that MT27 isolates from China are more likely to have been imported into China from other countries than to have arisen from closely related existing strains (*19*). All MT27 isolates were sensitive to erythromycin.

Many studies have shown that *ptxP* may play a role in pathogen adaptation. Strains with *ptxP1* were most common in the early WCV periods but were replaced by *ptxP3* strains in the WCV/ACV periods and ACV periods (*20*). Studies from Finland and Australia suggest that the increase of *ptxP3* strains may be associated with the resurgence of pertussis (*21*–*23*). The association between virulence-related genotype, erythromycin susceptibility, and MLVA types is in accordance with no resistance epidemic in other countries, including the United States (*24*), Australia (*22*), and many countries in Europe (*25*), because isolates from those countries mainly harbor *ptxP3* genes, and the prevalent MLVA types were MT27 and MT29.

Because the drug commonly used to treat erythromycin-resistant *B. pertussis* infection is sulfamethoxazole/trimethoprim (*26*), it is reassuring that this drug still shows powerful inhibition of these bacteria. Levofloxacin could be another choice for adult patients, and doxycycline might also be an alternative for adults with pertussis (*27*). Earlier, β-lactams were recommended for pediatric patients with pertussis (*28*,*29*). The MICs for β-lactams suggest that they could be used; however, their effectiveness for eliminating the bacteria was not comparable to that of macrolides. An explanation is that the local concentrations of β-lactams in the respiratory tract are insufficient (*30*).

It is noteworthy that there is no standard procedure for antimicrobial susceptibility testing for *B. pertussis*. We consider the striking macrolide resistance rates to be reliable because all macrolide-resistant isolates had erythromycin MICs >256 mg/L, no inhibition zone in Kirby-Bauer disk diffusion, and the A2047G mutation in 23S rRNA, which has been previously reported (*8**,**31*). To date, the Clinical and Laboratory Standards Institute and the European Committee on Antimicrobial Susceptibility Testing offer no suggestions for macrolides and other drugs that could be used to treat pertussis, even regarding appropriate use and dose. Development of standard methods for antimicrobial susceptibility testing of *B. pertussis* and monitoring the treatment effects of appropriate antimicrobial agents in vivo would be helpful. 

Our study had some limitations. First, all patients with clinically suspected pertussis were routinely subjected to nasopharyngeal swab culture in our study. However, the clinical diagnostic standard for pertussis is not specific in China and differs among age groups. Therefore, we did not set specific criteria for enrollment of patients, and the inclusion criteria were based on the subjective judgment of pediatricians. Second, our study was not based on the patient population (no sample collection and follow-up records with clinical information for patients in this study). Thus, we could not precisely evaluate the severity of individual cases and the association between disease severity and drug resistance or genotype. Third, although all clinical samples were collected during 2014–2016, the number of patients receiving treatment and the duration of recruitment in the included hospitals differed. Therefore, the number of isolates from the participating hospitals does not reflect the actual number of cases in the 17 provinces or municipalities. Fourth, before the patients visited the participating 4 hospitals, Beijing Children’s Hospital in particular, they had already received antimicrobial drugs. The high resistance rate may be associated with pretreatment with antimicrobial drugs and therefore may be overestimated. Alternatively, *B. pertussis* isolates with resistance were very common, which implies the failure of treatment with macrolides. Among study patients in Beijing Children’s Hospital, 23 were reexamined by culture after 2 weeks of macrolide treatment and 4 were still culture positive. It is conceivable that macrolide treatment could not eliminate the resistant bacteria. More rigorous comparisons should be conducted to interpret the clinical significance of resistance for this self-limiting disease. Last, for some objective reasons, the susceptibility against some drugs was tested in a subset of the present isolates (clarithromycin, azithromycin, and doxycycline in particular).

In conclusion, *B. pertussis* isolates genotyped as *ptxA1/ptxC1/ptxP1/prn1/fim2–1/fim3A/tcfA2* and highly resistant to erythromycin are widespread in China. The *ptxP3* strains sensitive to erythromycin were found mainly in southern China. Sulfamethoxazole/trimethoprim effectively treated pertussis caused by erythromycin-resistant *B. pertussis.* The MLVA profiles of *B. pertussis* isolates currently circulating in China differ from those circulating in other Western countries. 
